# Genetic analyses of DNA repair pathway associated genes implicate new candidate cancer predisposing genes in ancestrally defined ovarian cancer cases

**DOI:** 10.3389/fonc.2023.1111191

**Published:** 2023-03-08

**Authors:** Wejdan M. Alenezi, Caitlin T. Fierheller, Corinne Serruya, Timothée Revil, Kathleen K. Oros, Deepak N. Subramanian, Jeffrey Bruce, Dan Spiegelman, Trevor Pugh, Ian G. Campbell, Anne-Marie Mes-Masson, Diane Provencher, William D. Foulkes, Zaki El Haffaf, Guy Rouleau, Luigi Bouchard, Celia M. T. Greenwood, Jiannis Ragoussis, Patricia N. Tonin

**Affiliations:** ^1^ Department of Human Genetics, McGill University, Montreal, QC, Canada; ^2^ Cancer Research Program, Centre for Translational Biology, The Research Institute of McGill University Health Centre, Montreal, QC, Canada; ^3^ Department of Medical Laboratory Technology, Taibah University, Medina, Saudi Arabia; ^4^ McGill Genome Centre, McGill University, Montreal, QC, Canada; ^5^ Lady Davis Institute for Medical Research of the Jewish General Hospital, Montreal, QC, Canada; ^6^ Cancer Genetics Laboratory, Peter MacCallum Cancer Centre, Melbourne, VIC, Australia; ^7^ Princess Margaret Cancer Centre, University Health Network, Toronto, ON, Canada; ^8^ Montreal Neurological Institute, McGill University, Montreal, QC, Canada; ^9^ Department of Medical Biophysics, University of Toronto, Toronto, ON, Canada; ^10^ Ontario Institute for Cancer Research, Toronto, ON, Canada; ^11^ Sir Peter MacCallum Department of Oncology, University of Melbourne, Melbourne, VIC, Australia; ^12^ Centre de recherche du Centre hospitalier de l’Université de Montréal and Institut du cancer de Montréal, Montreal, QC, Canada; ^13^ Departement of Medicine, Université de Montréal, Montreal, QC, Canada; ^14^ Division of Gynecologic Oncology, Université de Montréal, Montreal, QC, Canada; ^15^ Department of Medical Genetics, McGill University Health Centre, Montreal, QC, Canada; ^16^ Department of Medicine, McGill University, Montreal, QC, Canada; ^17^ Gerald Bronfman Department of Oncology, McGill University, Montreal, QC, Canada; ^18^ Service de Médecine Génique, Centre Hospitalier de l’Université de Montréal, Montreal, QC, Canada; ^19^ Department of Biochemistry and Functional Genomics, Université de Sherbrooke, Sherbrooke, QC, Canada; ^20^ Department of Medical Biology, Centres intégrés universitaires de santé et de services sociaux du Saguenay-Lac-Saint-Jean hôpital Universitaire de Chicoutimi, Saguenay, QC, Canada; ^21^ Centre de Recherche du Centre hospitalier l’Université de Sherbrooke, Sherbrooke, QC, Canada; ^22^ Department of Epidemiology, Biostatistics and Occupational Health, McGill University, Montreal, QC, Canada

**Keywords:** germline variants, familial ovarian cancer, cancer predisposing genes, whole exome sequencing, DNA repair pathways, Genetic drift

## Abstract

Not all familial ovarian cancer (OC) cases are explained by pathogenic germline variants in known risk genes. A candidate gene approach involving DNA repair pathway genes was applied to identify rare recurring pathogenic variants in familial OC cases not associated with known OC risk genes from a population exhibiting genetic drift. Whole exome sequencing (WES) data of 15 OC cases from 13 families tested negative for pathogenic variants in known OC risk genes were investigated for candidate variants in 468 DNA repair pathway genes. Filtering and prioritization criteria were applied to WES data to select top candidates for further analyses. Candidates were genotyped in ancestry defined study groups of 214 familial and 998 sporadic OC or breast cancer (BC) cases and 1025 population-matched controls and screened for additional carriers in 605 population-matched OC cases. The candidate genes were also analyzed in WES data from 937 familial or sporadic OC cases of diverse ancestries. Top candidate variants in *ERCC5*, *EXO1*, *FANCC, NEIL1* and *NTHL1* were identified in 5/13 (39%) OC families. Collectively, candidate variants were identified in 7/435 (1.6%) sporadic OC cases and 1/566 (0.2%) sporadic BC cases versus 1/1025 (0.1%) controls. Additional carriers were identified in 6/605 (0.9%) OC cases. Tumour DNA from *ERCC5, NEIL1* and *NTHL1* variant carriers exhibited loss of the wild-type allele. Carriers of various candidate variants in these genes were identified in 31/937 (3.3%) OC cases of diverse ancestries versus 0-0.004% in cancer-free controls. The strategy of applying a candidate gene approach in a population exhibiting genetic drift identified new candidate OC predisposition variants in DNA repair pathway genes.

## Introduction

Since the identification of *BRCA1* (1) and *BRCA2* (2) as breast cancer (BC) and ovarian cancer (OC) predisposing genes, which are involved in the homologous recombination (HR) DNA repair pathway (3), no other major high risk gene has been reported to account for the remaining familial cancer cases found to be negative for germline pathogenic variants (PVs) in these genes (4). Carriers of PVs in *MLH1, MSH2*, *MSH6* or *PMS2*, genes involved in the mismatch repair (MMR) pathway (3), have also been shown to have a significantly increased lifetime risk of developing OC (5, 6) often associated with hereditary non-polyposis colorectal cancer syndrome families (7). However, carriers of PVs in MMR genes are very rare accounting for fewer than 1% of sporadic OC cases, which is significantly lower than the 5-15% carrier frequency of PVs in *BRCA1* and *BRCA2*, depending on the population studied (8). Carriers of PVs in relatively new OC predisposing genes have been reported such as *RAD51C* (9), *RAD51D* (10) and *BRIP1* (11), genes also involved in the HR DNA repair pathway (3). The carrier frequency of PVs in each of these genes combined is estimated to be less than 2% of sporadic OC cases (8, 12–14). PVs in other DNA repair genes such as *PALB2* (15–19), *CHEK2* (16, 20) and *ATM* (16, 17), all associated with BC risk, were recently associated with OC, though risk has yet to be established. Other genes also playing a role in various DNA repair pathways have been proposed as candidate OC risk genes such as *FANCM* (15, 21), *POLE* (22), *MRE11* (17, 23), *RAD1* (17) and *FANCI* (24), and collectively, the frequency of carriers of PVs in these genes are also low relative to *BRCA1* and *BRCA2* carriers. Thus, research has consistently shown that a candidate gene approach investigating DNA pathway genes has successfully identified new and candidate OC predisposing genes (25, 26), though it is expected that the carrier frequency is significantly lower relative to carriers harbouring PVs in *BRCA1* and *BRCA2*.

Defining the contribution of moderate- to high-risk genes in OC remains a challenge as it is not clear that all monogenic cancer predisposing genes have been identified for this genetically heterogeneous disease (4). Based on the family history of cancer and the population investigated, the proportion of OC families known to be negative for *BRCA1* or *BRCA2* PVs has a wide range of approximately 15-65% (8, 27). Indeed, a recent whole exome sequencing (WES) study of familial and sporadic OC cases revealed significant heterogeneity of candidate OC risk genes, representing diverse functional pathways with relatively few involved in the approximately 200 investigated DNA repair genes (17). However, this study focused only on investigating rare, protein-coding loss-of-function (LoF) variants (17). As there are at least 400 known or putative genes that are directly or indirectly involved in repairing DNA (3, 28–32), it is plausible that PVs (LoF or missense) in genes not previously investigated in OC could be associated with OC risk that have yet to be identified. As carriers of new candidate variants are likely to be rare, identifying them will be challenging.

We have proposed a strategy for identifying candidate variants in new cancer predisposing genes that involves the investigation of cancer families from populations exhibiting genetic drift (33). Over time, rare PVs in such populations could attain disproportionally high carrier frequencies of rare risk variants relative to the general population (34, 35). For example, *PALB2* (36) and *BRIP1* (11) were discovered as BC and OC predisposing genes by investigating cancer families and cases from the Finnish and Icelandic populations, respectively, both populations exhibiting genetic drift. Our research of French Canadians (FC) from the Quebec population of Canada, identified *RECQL* (37) and *FANCI* (24) as new candidate BC or OC predisposing genes, respectively. Genetic drift in the FCs of Quebec has been attributed to common ancestors as a result of the geographic isolation and multiple waves of expansion of European settlers from France since 1608 (33–35, 38, 39). Investigating these populations facilitates the characterization of deleterious variants in known or candidate cancer predisposing genes as all types of variants could be investigated and not only LoF variants (33). A small number of PVs in *BRCA1* and *BRCA2* (40, 41) and one in each of *PALB2* (42), *RAD51C* (43) and *RAD51D* (43, 44) have been shown to be frequently occurring in FC OC and/or BC cases versus population-matched controls. Specific PVs in *MLH1* (45)*, MSH2* (45) and *MSH6* (46) have also been reported in FCs in the context of hereditary non-polyposis colorectal cancer.

We recently reported that not all remaining *BRCA1* and *BRCA2* negative families with at least two close relatives with OC from the FC population of Quebec by WES analysis were due to PVs in *RAD51C*, *RAD51D* or *BRIP1* approximating that 40% of such cases are unaccounted for by known or emerging OC predisposing genes (33, 43, 47). Also, we reported that likely pathogenic variants (LPV) in *FANCI*, a proposed new OC predisposing gene from the Fanconi anemia (FA) pathway, were rarely implicated in familial and sporadic OC cases in this population (24). We posit that DNA repair pathways genes have not been fully explored as candidate OC risk genes. In this study, we report the identification of candidate LPVs in DNA repair pathway genes that were identified by applying a candidate gene approach focusing on an extensive list of 468 DNA repair pathway genes in available WES data derived from the germline of FC familial OC cases. Candidate variants prioritized based on our bioinformatic analyses were selected for targeted genotyping in larger groups of defined FC cases to determine carrier frequencies in 1212 FC and 937 non-FC familial and sporadic OC cases and 1025 population-matched controls. Available tumour DNA from our FC carriers also was investigated for loss of the wild-type allele of candidate loci.

## Materials and methods

### Study participants

**Figure 1:**
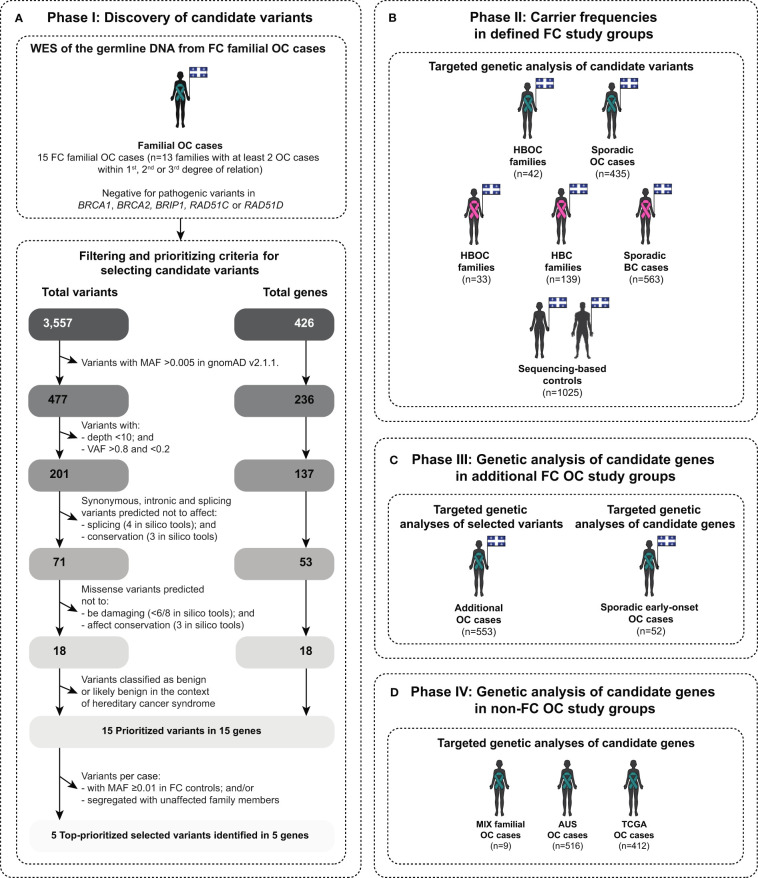
Scheme describing different phases of this study in identifying and evaluating candidate variants in genes involved in various DNA repair pathways. The diagram illustrates: **(A)** study phase I for identifying candidate variants by applying a candidate gene approach of known or putative DNA repair genes (see **Table S2**) on peripheral blood lymphocytes (PBL) DNA from familial ovarian cancer (OC) cases of French Canadians (FC) of Quebec by whole exome sequencing (WES) and bioinformatic analyses (see **Table S1**); **(B)** study phase II for determining the carrier frequencies of the topprioritized candidate variants in FC familial and sporadic OC and BC cases, including hereditary breast and ovarian cancer (HBOC) syndrome and hereditary breast cancer (HBC) syndrome families, and population-matched controls by targeted genetic analyses (see **Table S1**); **(C)** study phase III for identifying additional carriers in FC OC cases by targeted genetic analyses (see **Table S1**); and **(D)** study phase IV for identifying candidate variants in the identified candidate DNA repair genes from phase I in non-FC OC cases, mainly of European origin, by targeted genetic analyses: (MIX, mixed ethnicity; AUS, Australian; and TCGA, The Cancer Genome Atlas) (see **Table S1**). Teal ribbon signifies women with OC and pink ribbon signifies women with BC, and diagrams contain the provincial flag of Quebec, Canada denoting the geographic ascertainment of cases and controls. MAF, Minor allele frequency; and VAF, Variant allele frequency.

FC cancer cases and controls are described in **Figure 1** and **Table S1**. For clarity, cases diagnosed with primary fallopian tubes or peritoneal cancers were included as cases in recognition of common aetiology with OC, which are associated with hereditary cancer risk factors (48, 49). For the discovery of new candidate OC risk variants (study phase I; **Figure 1A**), WES data from peripheral blood lymphocytes (PBL) DNA was available from 15 OC cases from 13 families, each family having at least two first-, second- or third-degree relatives with OC. These cases were confirmed negative for PVs in the known OC risk genes: *BRCA1*, *BRCA2, BRIP1, RAD51C* or *RAD51D* as previously reported (47, 43). This group includes three index cases harbouring a LPV in *FANCI* c.1813C>T; p.Leu605Phe (24). As *FANCI* remains a candidate OC predisposing gene requiring further independent studies, we did not exclude *FANCI* variant carriers from any of our study groups for our investigation.

Targeted analyses of the candidate variants was performed to determine their carrier frequencies (study phase II; **Figure 1B**) on the PBL DNA from FC OC cases, regardless of their carrier status for *BRCA1* and *BRCA2* PVs, from 42 hereditary breast and ovarian cancer (HBOC) syndrome families having one OC and at least two BC cases in the same familial branch and 435 sporadic OC cases not selected for age at diagnosis with the disease or for family history of any cancers. Genetic data was available from 1025 population-matched controls provided by three independent biobanks as previously described (43). As known OC predisposing genes are also involved in BC risk (nccn.org/guidelines/category_2), targeted analyses of the candidate variants was also performed on the PBL DNA from FC BC cases, regardless of their *BRCA1* and *BRCA2* PV carrier status, from 33 HBOC families, 139 hereditary breast cancer (HBC) syndrome families having at least three close relatives with BC within first-, second- or third-degree of relationship from the same familial branch and 563 sporadic BC cases not selected for age at diagnosis with the disease or for family history of any cancers.

Targeted analyses of PBL DNA from additional OC cases was performed to identify more OC carriers of our candidate variants (study phase III; **Figure 1C**). These groups were comprised of: 52 sporadic early-onset cases diagnosed with high-grade serous ovarian carcinoma (HGSC) before the age of 50 years who tested negative for PVs in *BRCA1* or *BRCA2*; and 553 OC cases, regardless of their *BRCA1* and *BRCA2* PV carrier status and not defined by any criteria as previously described in this study (43).

The majority of FC cancer cases self-reported FC ancestry of Quebec as described previously (24, 40, 41, 43, 47, 50–56). FC controls from Université de Sherbrooke-The Genetics of Glucose Regulation in Gestation and Growth (Gen3G) (57) and McGill University-Montreal Neurological Institute (MNI) (58) biobanks self-reported FC ancestry as described previously (43). FC controls from CARTaGENE biobank (cartagene.qc.ca) were born in the province of Quebec, reported being FC ancestry, having parents and all four grandparents born in Canada and French as first language learned as described previously (43, 59).

The cancer cases and controls not selected for being of FC ancestry of Quebec, mainly of European ancestry are referred to as non-FC groups in this study, were available from different resources. Genetic analyses to determine the spectrum and prevalence of candidate variants in genes that were identified in the study phase I were performed (study phase IV; **Figure 1D**) on available genetic data derived from PBL DNA from three independent groups with OC: 9 OC cases from 7 families with at least two close relatives with OC (MIX familial OC cases) (47); 516 OC familial or sporadic cases from the Australian population (AUS OC cases) (17) and 412 OC cases as part of The Cancer Genome Atlas (TCGA) Pan-Cancer Atlas project (not selected for ethnicity) (60) and cancer-free controls as part of the Genome Aggregation Database (gnomAD) v2.1.1. (61). The gnomAD v2.1.1. controls were also used to filter common variants as part of study phase I (**Figure 1A** and **Table S1**) which is described in the following section.

All biological samples, clinico-pathological, pedigree and relevant medical genetic information from the cancer cases and control groups that were investigated in this study are from biobanks where participants had been recruited in accordance with ethical guidelines of the biobanks respective Institutions Research Ethics Boards as described in **Table S1**. Where applicable, samples were anonymized at source by the providers and were assigned a unique identifier (PT followed by four digits) to further protect their identity. This project was conducted with approval and in accordance with the guidelines of The McGill University Health Centre Research Ethics Board (MP-37-2019-4783).

### Identifying and selecting for top candidate variants in FC cancer cases

For phase I of the study (**Figure 1A**), WES data was available from PBL DNA from 15 OC index cases from 13 cancer families that had at least one first-, second- or third-degree relative from the same familial branch with OC, and were confirmed being negative for PVs in *BRCA1*, *BRCA2, BRIP1, RAD51C* or *RAD51D* by WES analyses (43, 47). WES had been subjected to a customized bioinformatics pipeline for germline variant calling at the McGill Genome Center as previously reported by our group (24, 43). In brief, NimbleGen SeqCap^®^ EZ Exome v3.0 library kit (Roche, US), followed by paired-end sequencing on different Illumina HiSeq platforms was performed. Reads were aligned to the human reference genome assembly GRCh37/hg19 using Burrows-Wheeler aligner v0.7.17, followed by PCR deduplication using Picard v2.9.0. Realignment around small insertions and deletions was performed, and germline variants were called using HaplotypeCaller using Genome Analysis Toolkit (GATK) v3.5. Variants were then filtered for base sequencing quality score ≥30 and annotated using Ensembl Variant Effect Predictor (VEP) and GEMINI v0.19.1.

Using a candidate gene approach, a curated list of 468 known or putative DNA repair genes (28–32) (ebi.ac.uk/QuickGO/term/GO:0006281) (**Table S2**) were investigated for candidate PVs in WES data from selected index OC cases (**Figure 1A**). Variants identified in these DNA repair genes were extracted from the annotated variant call format (VCF) files from the index OC cases (**Figure 1A** **)**. Variants with minor allele frequency (MAF) >0.005 in gnomAD v2.1.1. (61, 62), with total low coverage <10 reads and/or those with variant allele frequency (VAF) <0.2 and >0.8 were filtered out and retained variants were subjected for further prioritization and selection. These thresholds have been tested previously under the assumption that new variants follow an autosomal dominant mode of inheritance (63). These variants were then verified by manual inspection in the aligned sequences in compressed binary alignment map (BAM) files by Integrative Genomics Viewer (IGV) v2.4.10. (64).

Top candidate variants were selected from this master list of variants for further analyses based on various prioritization criteria as shown in **Figure 1A**. First, we prioritized LoF variants (nonsense, frameshift and alternative splicing variants), inframe, missense and intronic variants, which were predicted to be conserved and damaging at the RNA or protein level by 15 selected in silico tools: (1) by at least one out of three prediction tools for conservation Genomic Evolutionary Rate Profiling v1.0 (GERP++ [score≥2.0]) (65), Phylogenetic P value of 100 vertebrates v4.2 (PhyloP 100 way [score ≥0.2]) (66) and PHAST Conservation of 100 vertebrates v4.2 (PhastCons 100 way [score ≥0.9]) (67); (2) by at least one out of four prediction tools for splicing Maximum Entropy Estimates of Splice Junction v2.0 (MaxEntScan) (68), two different Database Splicing Consensus Single Nucleotide Variant (dbscSNV) in silico tools: AdaBoost v4.0 (ADA [score ≥0.4]) and Random Forest v4.0 (RF [score≥0.4]) (69) and SpliceAI (score ≥0.4) (70); and (3) at least six out of eight prediction tools for damaging of protein function based on their best performance (71–74): Combined Annotation Dependent Depletion v1.4 (CADD [Phred score ≥20]) (75), Eigen (score ≥0.0) (76), Meta-analytic Logistic Regression v4.2 (MetaLR [score ≥0.5]) (77), Meta-analytic support Vector Machine v4.2 (MetaSVM [score ≥0.0]) (77), MetaRNN 4.2 (score ≥0.5) (78), Rare Exome Variant Ensemble Learner v4.2 (REVEL [score ≥0.5)) (79), Variant Effect Scoring Test v4.2 (VEST [score ≥0.5]) (80) and Protein Variation Effect Analyzer v4.0 (PROVEAN v4.0 [score ≤−2.5]) (81). Then, the variants having a clinical classification as benign or likely benign in the context of hereditary cancer syndromes in ClinVar (82, 83) and/or American College of Medical Genetics and Genomics (ACMG) guidelines (84, 85) were given a lower priority for further investigation.

The remaining prioritized variants were then subjected to further prioritization. Variants were surveyed in available genetic data generated from the germline of three FC study groups (**Table S1**): (1) WES data from the germline of 52 sporadic early-onset OC cases negative for PVs in *BRCA1* and *BRCA2* (43, 47); and (2) sequencing-based (WES or whole genome sequencing [WGS]) data and/or genotyping-based data from 1025 FC controls (43). Then, the variants were subjected for further selection and characterization for genetic analyses.

Selected top candidate variants were verified in the PBL DNA by bidirectional Sanger sequencing using customized primers (available upon request) performed at the McGill Genome Center as described previously (86, 43, 47; 24, 33). Sequencing chromatograms were visually inspected for variant heterozygosity using 4Peaks v1.8. (nucleobytes.com/4peaks/) (The Netherlands Cancer institute, Amsterdam, The Netherlands).

### Determining carrier frequencies of selected candidate variants in FC cancer cases and controls

Selected top candidate variants were investigated for carrier frequencies in defined FC study groups (study phase II) comprised of 42 index OC and 33 index BC cases from 75 HBOC families, 139 index BC cases from 139 HBC families, 435 sporadic OC cases and 563 sporadic BC cases (**Figure 1B** and **Table S1**). PBL DNA from index cases were genotyped using customized TaqMan^®^ (87), Sequenom iPLEX^®^ Gold (88) or Fluidigm^®^ SNP Type^™^(89) genotyping assays (primers available upon request) as described previously (24, 43, 90). Tumour DNA samples from the index case were genotyped where PBL DNA was no longer available from the biobank. Carriers of candidate variants were verified by bidirectional Sanger sequencing of PBL DNA as described above. Selected candidate variants were also investigated for carrier frequency in population-matched controls by surveying 1025 available sequencing-based data sets (**Table S1**) and/or 8493 single nucleotide polymorphism (SNP) genotyping-based data sets as previously described (43). For probes of variants not presented on the SNP array, pre-phasing and imputation were performed as described previously (24, 43).

Pair-wise comparisons were performed of carrier frequencies of candidate variants in different FC cancer groups versus sequencing-based controls. Two-tailed Fisher’s exact test was used to compare carrier frequencies in the cancer versus control groups where un-adjusted *P* values <0.05 for multiple testing was considered significant.

### Targeted genetic analyses of selected candidate variants or genes in FC cancer cases

To further characterize our candidate variants and genes in a population exhibiting genetic drift, we investigated carrier status in additional OC cases from the FC population. Selected top candidate variants were investigated (study phase III) in 52 sporadic early-onset FC HGSC cases and in an additional 553 FC OC cases by surveying available genetic data or targeted genotyping of PBL DNA (**Figure 1C** and **Table S1**). We also investigated other variants in our gene candidates that met our filtering and prioritizing criteria in the available WES data from the sporadic early-onset OC cases (**Figure 1C**).

### Loss of heterozygosity analyses of candidate genes loci in OC tumour DNA from FC candidate variant carriers

To investigate evidence for inactivation of candidate genes in cancer cells, we performed loss of heterozygosity (LOH) analysis of tumour DNA from variant carriers. Bi-directional Sanger sequencing of available tumour DNA was performed using customized primers (available upon request) as described above. Extracted DNA from fresh-frozen (FF) or histopathological sections from formalin-fixed paraffin-embedded (FFPE) tumour tissues were provided by the RRCancer biobank for DNA extraction and LOH analysis (Promega, Canada). Sequencing chromatograms were inspected for loss of the wild-type allele using 4Peaks v1.8. (nucleobytes.com/4peaks/) (The Netherlands Cancer Institute, Amsterdam, The Netherlands).

### Genetic analyses of candidate genes in non-FC cancer cases and controls

To further characterize the candidate variants and genes identified in our FC cancer cases, we investigated available genetic data from other populations that were not specifically selected for FC ancestry. The spectrum and prevalence of our candidate variants were investigated in genetic data from non-FC OC cases being predominantly of European ancestry and cancer-free controls were investigated for new variants in our candidate genes that met our filtering and prioritizing criteria (**Figure 1D** and **Table S1**). Variants were extracted from the annotated VCF files generated by WES data from the germline of: (1) 9 MIX familial OC cases; (2) 516 AUS OC cases; and (3) 412 OC cases from the Pan-Cancer TCGA project. Variants were extracted from the comma separated value (CSV) files downloaded directly from (https://gnomad.broadinstitute.org). All variants were annotated and subjected to our filtering and prioritizing criteria to identify candidate variants as described above (see **Figure 1A**).

### Genetic analyses for co-occurring rare pathogenic variants in known OC risk genes in FC and non-FC candidate variant carriers

We investigated available WES data from OC cases, to determine whether the identified carriers of candidate variants (regardless of the ethnicity and study phase in which they were identified) also harbour rare PVs or LPVs in known OC risk genes (n=11): *BRCA1* (NM_007294.4), *BRCA2* (NM_000059.4), *MLH1* (NM_000249.4), *MSH2* (NM_000251.3), *MSH6* (NM_000179.3), *PMS2* (NM_000535.7), *BRIP1* (NM_032043.3), *RAD51C* (NM_058216.3), *RAD51D* (NM_001142571.2), *PALB2* (NM_024675.4) and *ATM* (NM_000051.4) based on the National Comprehensive Cancer Network (NCCN) Clinical Practice in Oncology Guidelines 2022 (Version 2.2022) —Genetic/Familial High-Risk Assessment: Breast, Ovarian and Pancreatic (nccn.org/guidelines/category_2). Variants were extracted from the annotated VCF files from carriers and subjected to our filtering and prioritizing criteria as previously described (47).

## Results

### Prioritization and selection of candidate variants: Phase I

**Figure 2:**
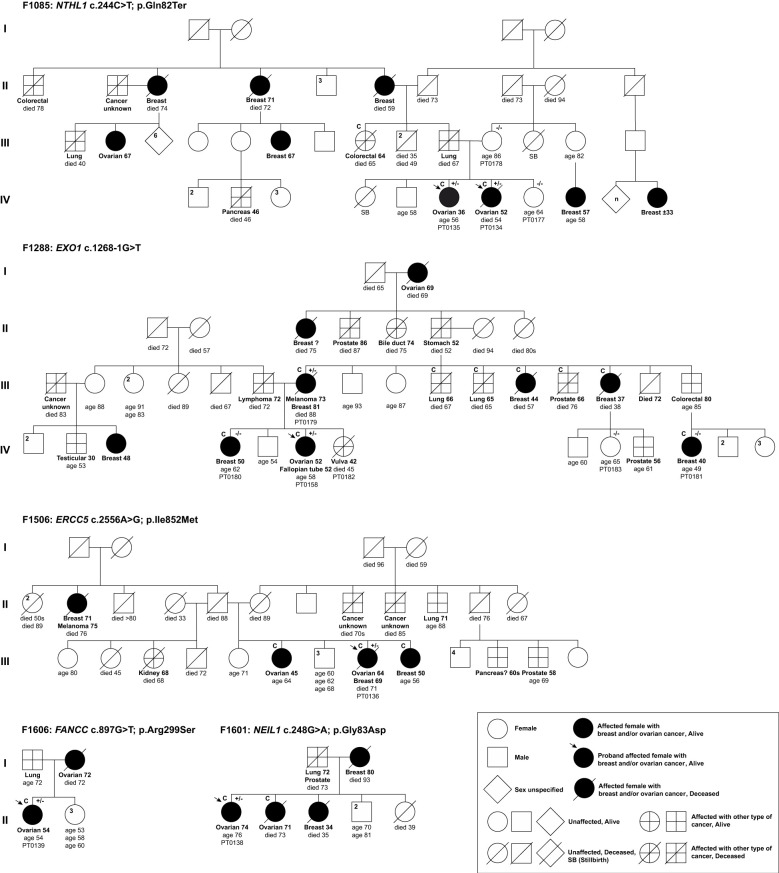
Pedigrees of index ovarian cancer cases harbouring candidate variants in DNA repair genes identified in phase I of the study. Selected top candidate variants were identified in 5 of 13 families having at least two or more OC cases. Anonymized pedigrees indicate carrier status of tested index case (arrow) and available family members denoted by plus (carrier) or minus (not a carrier) signs. All Index cases (arrow) were subjected to whole exome sequencing analyses (WES). All carriers were found in a heterozygous state. Age in years is shown at cancer diagnosis and death where applicable. Superscript C denotes histological subtypes that were confirmed by pathology reports or death certificates.

We first extracted variants identified in a curated list of 468 DNA repair genes (**Table S2**) from VCF files generated from WES data from the 15 index FC OC cases from 13 families. In these index cases, we identified a total of 3,557 variants in 426 of 468 DNA repair genes (**Figure 1**). Based on their rarity and variant quality, we retained a total of 201 variants in 137 of 426 DNA repair genes where each index case harboured 3 to 25 (median=15) variants. From this list of 201 variants, we prioritized candidates that were predicted to be conserved or damaging at the level of RNA or protein using our selected in silico tools; and those classified benign or likely benign in the context of hereditary cancers using ClinVar and/or ACMG guidelines were not pursued further. Using these criteria, we retained a total of 15 of the 201 variants, each of which was found in a different gene: 3 nonsense variants, 1 canonical alternative splicing variant, 1 inframe and 10 missense variants (**Table S3**). These variants were identified in 10 of the 15 index cases from 8 out of the 13 OC families (**Figures 2**, **S1**). One of these variants was identified in two OC cases from the same family, two variants were identified in two OC cases from the same family and the remaining 12 variants were identified in one index case from independent OC families (**Table S3**). Two cases harboured either three or four variants, while the remaining nine cases harboured one to two variants.

To select our top candidates for further analyses, we reviewed the individual context wherein the 15 variants were identified as shown in **Table S3** and **Figures 2**, **S1**. We estimated the allele frequencies of the 15 candidate variants in population-matched FC controls. Thereby, we did not pursue *ALKBH3* c.677A>G; p.Asn226Ser, which was identified in an index case (PT0136) from family F1506, as it has a MAF ≥0.01 in FC controls. We also excluded the missense variant in *DNA2* c.836C>T; p.Thr279Ile for further analyses as it was identified in the index case (PT0128) and four unaffected members of the family F694, and was not inherited from the affected mother with the family history of OC and other cancers (**Figure S1**). We did not pursue *RBBP8* c.1941T>G; p.Asp647Glu as it was not harboured by the other index OC case (PT0056) from the same family F1528 (**Figure S1**). Additional variants that were excluded for further analyses included: (1) *RHNO1* c.250C>T; p.Arg84Ter in the index OC case (PT0158) from family F1288; (2) *ATRX* c.4377_4379del; p.Glu1464del in one of the index OC case (PT0057) from family F1528 as they were classified as benign in ClinVar and by ACMG guidelines, and the latter as not being harboured by the other index OC case of the same family F1528 (**Figure S1**); (3) *SMARCA2* c.3265C>T in the index OC case (PT0128) from family F694; p.Arg1089Trp; and (4) *KMT2C* c.6916C>T; p.Pro2306Ser in the index OC case (PT0047) from family F1490 as variants in these genes are associated with non-cancer related syndromes (**Figure S1**). Heterozygous germline variants in *SMARCA2* are linked with Nicolaides-Baraitser syndrome (MIM: 601358), which is characterized by intellectual disability, seizures, limited to absence of speech ability, short stature, dysmorphic facial features and sparse hair (91, 92; 93–97); and heterozygous germline variants in *KMT2C* are linked with Kleefstra syndrome, type 2 (MIM: 617768), which is characterized by delayed psychomotor development, variable intellectual disability and mild dysmorphic features (98–101). A genotype-phenotype of heterozygous variants located within exon 15-25 of *SMARCA2*, which encodes the ATPase domain, have been recently reported that over 80% of these variants were *de novo* based on WES analyses of 80 cases in trios with Nicolaides-Baraitser syndrome that have been documented worldwide so far (92, 95, 97). Our *SMARCA2* c.3265C>T; p.Arg1089Trp has never been reported in the literature, but it is located in exon 23 that encodes the ATPase domain (97). Whereas, *KMT2C* c.6916C>T; p.Pro2306Ser was reported in the context of Kleefstra syndrome (100). Finally, we did not pursue: *RECQL5* c.918G>A; p.Met306Ile, *ASCC3* c.3808C>T; p.Arg1270Ter and *UBB* c.569C>A; p.Pro190His as all were harboured by the same index case (PT0139) from family F1606 that also harbouring *FANCC* c.897G>T; p.Arg299Ser as this variant is a plausible and intriguing candidate where *FANCC* has been reported as a candidate BC predisposing gene (102). Thus, *FANCC* c.897G>T; p.Arg299Ser and the remaining variants identified in *ERCC5* c.2556A>G; p.Ile852Met*, EXO1* c.1268-1G>T*, NEIL1* c.248G>T; p.Gly83Asp and *NTHL1* c.244C>T; p.Gln82Ter from our list of most promising variants were selected for further analyses as candidates.

### Characterization of the selected top candidate variants: Phase I

We selected five variants each identified in an OC family for further characterization and analyses (**Table 1** and **Figure 2**): a nonsense variant *NTHL1* c.244C>T; p.Gln82Ter, a canonical splicing variant *EXO1* c.1268-1G>T, an exonic splicing variant *FANCC* c.897G>T; p.Arg299Ser and two missense variants *ERCC5* c.2556A>G; p.Ile852Met and *NEIL1* c.248G>T; p.Gly83Asp. Both missense variants were predicted to affect amino acid residues that are located in catalytic domains of their respective proteins that are critical to the biological function of ERCC5 (108) and NEIL1 (109) in the HR, nucleotide excision repair (NER) and base excision repair (BER) pathways.

**Table 1:** Characteristics of top-prioritized candidate variants identified in familial French Canadian cases with ovarian cancer.

Genomic features (hg19/GRCh37)^1^					
Gene	*NTHL1*	*EXO1*	*FANCC*	*ERCC5*	*NEIL1*
Transcript	NM_002528.7	NM_130398.4	NM_000136.3	NM_000123.4	NM_024608.4
Cytoband	16p13.3	1q43	9q22.32	13q33.1	15q24.2
Genome change	g.2096239G>A	g.242035333G>T	g.97887467C>G	g.103520485A>G	g.75641494G>A
Coding change	c.244C>T	c.1268-1G>T	c.897G>T	c.2556A>G	c.248G>T
Protein change	p.Gln82Ter	–	p.Arg299Ser	p.Ile852Met	p.Gly83Asp
Allele frequencies in gnomAD^2^					
Non-cancer non-Finnish European	0.002 (235/118138)	–	8.8e-06 (1/113756)	9.74e-06 (1/102714)	0.001 (140/117290)
Clinical classification^3^					
ClinVar (number of submissions)	PV (6); VUS (1)	–	VUS (2)	–	–
ACMG guidelines (implemented rule)	PV (PVS1)	LPV (PVS1/PP3/PM2)	LPV (PVS1/PP3/PM2)	VUS (PP3/PM2)	LB (PP3/BP1)
Predictions by *in silico* tools^4^					
GERP++ v1.0	Conserved	Conserved	Conserved	Conserved	Conserved
PhyloP 100 way v4.2	Not conserved	Conserved	Not conserved	Not conserved	Conserved
PhastCons 100 way v4.2	Not conserved	Conserved	Not conserved	Conserved	Conserved
REVEL v4.2	–	–	Benign	Pathogenic	Pathogenic
MetaLR v4.2	–	–	Tolerated	Tolerated	Damaging
MetaSVM v4.2	–	–	Tolerated	Tolerated	Damaging
MetaRNN v4.2	–	–	Damaging	Damaging	Tolerated
CADD v1.6	Damaging	Damaging	Damaging	Damaging	Damaging
VEST v4.2	–	–	Damaging	Damaging	Damaging
EIGEN PC v4.2	–	–	Pathogenic	Pathogenic	Pathogenic
PROVEAN v4.2	–	–	Damaging	Damaging	Damaging
ADA v1.1	–	Affecting splicing	Affecting splicing	–	–
RF v1.1	–	Affecting splicing	Affecting splicing	–	–
MaxEntScan v2.0	–	Affecting splicing	Affecting splicing	–	–
SpliceAI	–	Affecting splicing	Affecting splicing	–	–

^1^ Annotation of candidate variants based on the National Center for Biotechnology Information (NCBI) - Reference Sequence (RefSeq) database (tark.ensembl.org/web/manelist/) (103); ^2^ Allele frequencies in the non-cancer, non-Finnish European controls from the Genome Aggregation Database (gnomAD) v2.1.1 database (gnomad.broadinstitute.org) (61); ^3^ Clinical classifications from ClinVar (ncbi.nlm.nih.gov/clinvar/) (82, 83) based on last revision in March 2022, and American College of Medical Genetics and Genomics (ACMG) guidelines (84, 104, 85, 105); ^4^ Applied in silico tools for conservation, damaging or affecting splicing selected based on their best performance (71, 74, 106, 107). Classification of variants by ACMG guidelines as: BP1: Benign Supporting Level 1; LB: Likely Benign; LPV: Likely Pathogenic Variant; PM2: Pathogenic Moderate Level 2; PP3: Pathogenic Supporting Level 3; PV: Pathogenic Variant; PVS1: Pathogenic Very Strong Level 1; VUS: Variant of Uncertain Significance; and (-): Not applicable/reported.

Except for *EXO1* c.1268-1G>T, which was not found in the gnomAD v2.1.1. database, all other candidate variants were found to have MAFs between 0.002 and 0.00001 in the non-cancer non-Finnish European populations with variation in these frequencies across populations of different ancestry groups (**Table S4**). The loci of all five candidate variants were predicted to be conserved by at least one of the selected in silico tools. The variants in *EXO1* and *FANCC* were predicted to affect splicing by all four selected in silico tools. The missense variants in *ERCC5* and *NEIL1* were predicted to be damaging by at least six selected in silico tools, including REVEL and VEST, which are two of the recently validated as top performing prediction in silico tools (73) (**Table S3**). Only *NTHL1* c.244C>T; p.Gln82Ter is classified as PV in ClinVar and by ACMG guidelines in the context of hereditary multi-cancer syndrome in an autosomal recessive mode of inheritance, and has recently been associated with BC risk in an autosomal dominant mode of inheritance (110, 111). Whereas, *FANCC* c.897G>T; p.Arg299Ser was classified as being of uncertain significance (VUS) in ClinVar in the context of FA, an autosomal recessive disorder (MIM: 227645) and as LPV by ACMG guidelines. As noted above, *FANCC* has been associated with BC predisposition in an autosomal dominant mode of inheritance (102). The remaining candidate variants have not been reported in ClinVar, but classified by ACMG guidelines as LPV for *EXO1* c.1268-1G>T, VUS for *ERCC5* c.2556A>G; p.Ile852Met and likely benign for *NEIL1* c.248G>T; p.Gly83Asp.

We genotyped PBL DNA samples from family members of the index carriers where possible to determine if the candidate allele segregated with disease (**Figure 2**). For family F1085, both the unaffected mother (PT0178) and sister (PT0177) of the index carrier cases did not carry *NTHL1* c.244C>T; p.Gln82Ter, suggesting that the variant allele may have been transmitted paternally. This observation is interesting as the paternal side of the family had numerous cancer cases including BC, OC, colorectal and pancreatic cancers. In family F1288, though the mother of the index *EXO1* c.1268-1G>T carrier case (PT0158) with BC and melanoma also carried the *EXO1* variant, her sibling (PT0180) and maternal female cousin (PT0181) both with BC were not carriers of the *EXO1* variant. These observations are interesting given the number of different types of cancer cases on the maternal side of the family. In family F1506, the index carrier harbouring *ERCC5* c.2556 A>G also had a remarkable family history of diverse cancer types, whereas the index carrier of *NEIL1* c.248G>T; c.1268-1G>T from family F1601 had a cancer family history consistent with HBOC syndrome. The index carrier of *FANCC* c.897G>T; p.Arg299Ser from family F1606 reported a mother with OC and a father with lung cancer.

### Identification of carriers of selected candidate variants in defined FC cancer study groups: Phase II

We genotyped or surveyed available genetic data of our candidate variants: *NTHL1* c.244C>T; p.Gln82Ter, *EXO1* c.1268-1G>T*, FANCC* c.897G>T; p.Arg299Ser, *ERCC5* c.2556A>G; p.Ile852Met and *NEIL1* c.248G>T; p.Gly83Asp in different FC OC and BC study groups and population-matched controls, regardless of their carrier status for *BRCA1* and *BRCA2* PVs (**Figure 1B** and **Table S1**). Carriers were identified in the sporadic OC study group with frequencies of 0.2% (1/435) for *EXO1* variant carriers and 0.5% (2/435) for carriers of each *NTHL1*, *ERCC5* or *NEIL1* variants, and one *NTHL1* carrier among sporadic BC cases (0.2%, 1/563). Carriers were not identified among index cases from HBOC and HBC families. *FANCC* variant carriers were not identified in any of these FC cancer study groups.

Carriers of all candidate variants in the FC controls are likely very rare as indicated by the observation that only one carrier was identified among 1025 FC sequencing-based controls (**Table 2**). Identifying a carrier of *NTHL1* c.224C>T; p.Gln82Ter was not surprising given the frequency of carriers of this variant = 0.002 in the non-cancer non-Finnish European population in gnomAD v2.1.1 (**Table S4**). Overall, the carrier frequencies of our candidates are higher in cancer groups relative to our population-matched controls though the results were not significant (**Table 2**).

**Table 2:** Carrier frequency of candidate variants in French Canadian cancer cases and controls.

Gene	Variant	Study groups	Cancer cases tested	Number of tested participants (or families) per study group	Number of variant carriers (%)	*p*-value
*NTHL1*	c.244C>T; p.Gln82Ter	HBOC families	OC	42 (42)	0	–
		Sporadic OC cases	OC	435	2/435 (0.5)	0.213
		HBOC families	BC	33 (33)	0	–
		HBC families	BC	139 (139)	0	–
		Sporadic BC cases	BC	563	1/563 (0.2)	1.000
		FC sequencing- based controls	–	1025	1/1025 (0.1)	–
*EXO1*	c.1268-1G>T	HBOC families	OC	42 (42)	0	–
		Sporadic OC cases	OC	435	1/435 (0.2)	0.298
		HBOC families	BC	33 (33)	0	–
		HBC families	BC	139 (139)	0	–
		Sporadic BC cases	BC	563	0	–
		FC sequencing- based controls	–	1025	0	–
*FANCC*	c.897G>T; p.Arg299Ser	HBOC families	OC	42 (42)	0	–
		Sporadic OC cases	OC	435	0	–
		HBOC families	BC	33 (33)	0	–
		HBC families	BC	139 (139)	0	–
		Sporadic BC cases	BC	563	0	–
		FC sequencing- based controls	–	1025	0	–
*ERCC5*	c.2556A>G; p.Ile852Met	HBOC families	OC	42 (42)	0	–
		Sporadic OC cases	OC	435	2/435 (0.5)	0.213
		HBOC families	BC	33 (33)	0	–
		HBC families	BC	139 (139)	0	–
		Sporadic BC cases	BC	563	0	–
		FC sequencing- based controls	–	1025	0	–
*NEIL1*	c.248G>T; p.Gly83Asp	HBOC families	OC	42 (42)	0	–
		Sporadic OC cases	OC	435	2/435 (0.5)	0.213
		HBOC families	BC	33 (33)	0	–
		HBC families	BC	139 (139)	0	–
		Sporadic BC cases	BC	563	0	–
		FC sequencing- based controls	–	1025	0	–

Two-tailed p-values (not adjusted for multiple testing) calculated using Fisher’s exact test in pair-wise comparisons between variant carriers in cancer study groups and population-matched controls. BC, Breast cancer; HBC, Hereditary breast cancer syndrome; HBOC, Hereditary breast and ovarian cancer syndrome; OC, Ovarian cancer; and (-), Not applicable.

We investigated our variants in 8493 non-cancer SNP array genotyping-based controls from cancer-free FC population (see **Table S1**). None of the probes for variants in *EXO1, FANCC* and *ERCC5* were represented on any of the SNP arrays, nor was imputation possible as they were not represented in the Haplotype Reference Consortium (HRC.r1) release panel (43). However, we were able to determine the carrier frequency of the *NTHL1* (0.2%, 19/8493) and *NEIL1* (0.3%, 24/8493) variants (**Table S5**). The frequencies of these variants are consistent with those in the non-cancer non-Finnish European population in gnomAD v2.1.1. (**Table S4**), though we did not identify any carriers among the FC controls harbouring both *NTHL1* and *NEIL1* candidate variants.

### Genetic analyses of other FC OC cases identified additional carriers of candidate variants: Phase III

Given genetic drift exhibited by the FC population that may result in higher frequency of candidate variant carriers with OC (34, 35, 43), we genotyped the germline of PBL DNA from additional 553 FC OC cases, which were recruited to the biobank but did not meet our criteria for the abovementioned defined OC study groups, and surveyed WES data available from 52 early-onset OC cases (**Figure 1C** and **Table S1**). We identified a total of six OC cases harbouring *NTHL1* c.244C>T (n=1), *EXO1* c.1268-1G>T (n=1), *FANCC* c.897G>T (n=1) and *NEIL1* c.248G>A (n=3) (**Table S6**).

### Genetic analyses of sporadic early-onset FC OC cases identified other variants in our candidate genes: Phase III

Given the genetic heterogeneity observed in the FC population for rare PVs identified in *BRCA1, BRCA2*, *RAD51C* and *RAD51D* (33, 43, 44), we surveyed WES data available from 52 early-onset FC OC cases diagnosed at less than 50 years of age (**Figure 1C** and **Table S1**). The rationale for investigating this group is based on the plausibility that carriers of some of the known OC predisposing genes are more likely to develop OC before age of 60 as it is the median age of diagnosis of this disease in the general population with OC (112, 113). We identified a carrier of a rare variant in *NEIL1* c.569C>A; p.Pro276His that met our filtering and prioritizing criteria (**Table S6**). We genotyped this variant in our defined FC cancer study groups and controls, and we did not identify any other carriers of this *NEIL1* variant or in any of the additional 553 FC OC cases. We could not determine the variant carrier frequency in the 8493 genotyping-based FC cancer-free controls as it was not represented on the SNP array, and we could not impute this variant as it was not available in the HRC.r1 haplotype reference panel.

### Evidence of loss of the wild-type allele in tumour DNA from carriers of candidate variants

**Figure 3:**
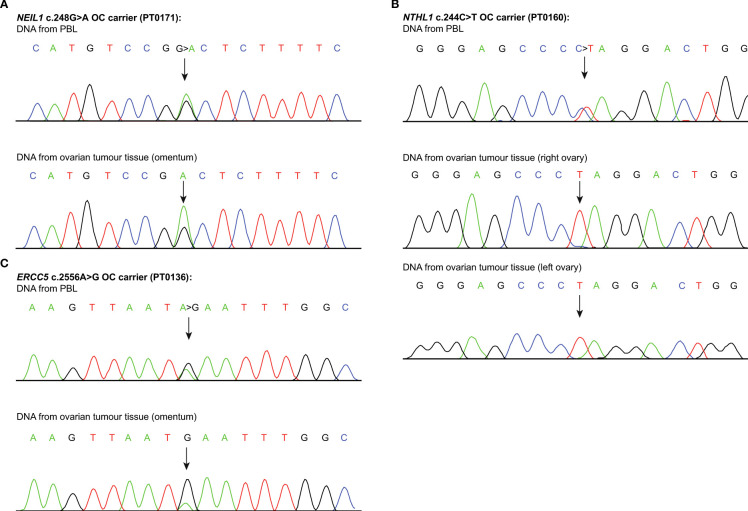
Loss of heterozygosity analyses of candidate genes loci. Sanger sequencing chromatograms showing loss of heterozygosity (LOH) analyses of the candidate variants (see **Table S7**), in genomic peripheral blood lymphocyte (PBL) DNA, ovarian tumour tissue DNA from carriers of **(A)** *NEIL1* c.248G>T; p.Gly83Asp (PT0171); **(B)** *NTHL1* c.244C>T; p.Gln82Ter (PT0160); and **(C)** *ERCC5* c.2556A>G; p.Ile852Met (PT0136). Each variant is indicated by an arrow. One example of such genetic event per candidate variant carrier is shown.

As known OC risk genes behave as tumour suppressors where there is loss of the gene function in tumours is be expected (114), we performed LOH analyses to investigate one of the classical mechanisms of inactivation of the loci of our candidate genes: *ERCC5*, *EXO1*, *FANCC*, *NEIL1* or *NTHL1*. We were able to perform LOH analyses on OC tumour DNA from our FC carriers of the candidate variants where possible due to availability from the RRCancer biobank as follows: *NTHL1* c.244C>T; p.Gln82Ter (n=4), *FANCC* c.897G>T; p.Arg299Ser (n=1), *ERCC5* c.2556A>G; p.Ile852Met (n=2) and *NEIL1* c.248G>T; p.Gly83Asp (n=3) (**Table S7**). Chromatograms of bidirectional Sanger sequencing of OC tumour DNA and case matched normal were inspected for allelic content. We observed partial or complete loss of the wild-type alleles in the tumour DNA from two *NEIL1* variant carriers, two *NTHL1* variant carriers and one *ERCC5* variant carrier (**Table S7**); chromatogram of one example is shown in **Figure 3**. Moreover, we observed partial or complete loss of the wild-type alleles in the tumour DNA from the left and the right ovaries from both *NTHL1* variant carriers having bilateral OC. There was no clear evidence for loss of the wild-type allele in the remaining samples from tumour DNA from carriers of *FANCC* or *EXO1*. However, loss of the variant allele was observed in the tumour DNA from *FANCC* and *EXO1* variant carriers (**Table S7**).

### Genetic analyses of non-FC cases identified other candidate variants in our gene candidates: Phase IV

**Figure 4:**
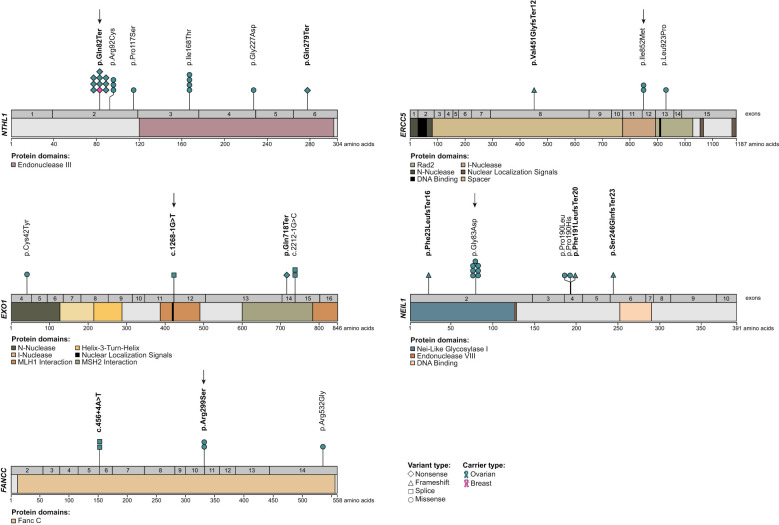
Location of candidate variants in *NTHL1*, *EXO1*, *FANCC*, *ERCC5* and *NEIL1* identified in all study groups. The coding regions and protein domains of candidate genes *NTHL1* (NM_002528.7), *EXO1* (NM_130398.4), *FANCC* (NM_000136.3), *ERCC5* (NM_000123.4) and *NEIL1* (NM_024608.4), based on NCBI RefSeq transcripts (tark.ensembl.org/web/manelist/) (103), were annotated for the location of candidate variants. Variants classified as PV or LPV are bolded and those identified in French Canadian ovarian cancer cases each are indicated with an arrow.

To determine the relevance of our candidate genes to OC in non-FC populations, we investigated the spectrum and prevalence of rare variants in our candidate genes in genetic data from three defined non-FC study groups (**Figure 1D** and **Table S1**). We applied our filtering and prioritizing criteria to WES data that was available from the germline of PBL DNA from: (1) 9 index OC cases from MIX familial OC cases; (2) 516 index AUS OC cases from HBOC and sporadic disease; and (3) 412 OC cases from Pan-Cancer – TCGA. In these study groups, we identified: one of the 9 MIX familial OC cases (11.1%) harbouring a *NTHL1* variant; 17 of 516 AUS OC cases (3.3%) harbouring 11 variants in *NTHL1* (n=6), *NEIL1* (n=3), *ERCC5* (n=1) and *EXO1* (n=1); 12 of 412 Pan-Cancer – TCGA OC cases (2.9%) harbouring 10 variants in *NEIL1* (n=4), *EXO1* (n=2), *FANCC* (n=2), *NTHL1* (n=1) and *ERCC5* (n=1) (**Table S6** and **Figure 4**). Noteworthy, the frequency of all LoF rare (MAF ≤0.005) variants in these genes in the cancer-free gnomAD v2.1.1 controls as follows: 112/1,563 (0.07) in *ERCC5*; 105/1,207 (0.09) in *EXO1*; 87/943 (0.09) in *FANCC*; 85/811 (0.1) in *NEIL1* and 47/629 (0.07) in *NTHL1*. Collectively, candidate variants we identified in cancer cases are comprised of three nonsense, four frameshift, three alternative splicing and nine missense variants. Variants in and *NEIL1* (n=3), *EXO1* (n=2), *NTHL1* (n=2), *ERCC5* (n=1) and *FANCC* (n=1) were LoF variants and classified as PVs or LPVs in ClinVar and/or by ACMG guidelines. The remaining variants were missense predicted to be PV or LPVs by our set of in silico tools. Some of these variants were those already identified in our FC study groups: three of 516 (0.6%) AUS OC cases and two of 412 (0.5%) Pan-Cancer – TCGA OC cases carried *NTHL1* c.244C>T; p.Gln82Ter, while one each of 516 (0.2%) AUS OC cases and 412 (0.2%) Pan-Cancer – TCGA OC cases carried *NEIL1* c.248G>A; p.Gly83Asp.

We identified one Pan-Cancer – TCGA OC carrier of a synonymous variant in *NEIL1* c.159C>T; p.Gly53Gly that was predicted to affect splicing using SpliceAI (115) that may result in gain of a new donor splice site.

### Most candidate variant carriers do not harbour co-occurring pathogenic variants in known OC predisposing genes

We investigated whether OC carriers harbouring any of our candidate variants may also harbour PVs in known OC predisposing genes (n=11) (nccn.org/guidelines/category_2) (**Tables S8**, **S9**). Only one of the 15 FC OC carriers investigated carried both *NEIL1* c.248G>A; p.Gly83Asp (PT0175) and *BRCA1* c.5102_5103del; p.Leu1701GlnfsTer14. This *BRCA1* variant is one of the most frequently occurring PVs in OC and BC cases from the FC population (33, 40, 41, 116).

Regarding non-FC OC carriers, one of the 17 OC AUS carriers of *NEIL1* c.248G>A; p.Gly83Asp (PT0314) also harboured a variant in a known OC risk gene *RAD51C* c.145+1_145+2insC, which was classified as PV by ACMG guidelines (**Table S9**) and was reported previously in an investigation of this study group (17). Two of the 13 carriers of our candidate variants from OC Pan-Cancer – TCGA project cases, harboured variants in known OC risk genes: a carrier of *NTHL1* c.244C>T; p.Gln82Ter (PT0261) also harboured *BRCA2* c.5065_5066insA; p.Ala1689AspfsTer6 and a carrier of *EXO1* c.2152C>T; p.Gln718Ter (PT0263) also harboured *BRCA2* c.1029del; p.Lys343AsnfsTer6. Both *BRCA2* variants have been classified as PVs in the ClinVar database and by ACMG guidelines.

## Discussion

Our investigation of potentially deleterious variants in 468 genes that play a direct or associated role in various DNA repair pathways in the FC population exhibiting genetic drift identified LoF and potentially deleterious missense variants as candidates for OC predisposition in *ERCC5*, *EXO1*, *FANCC*, *NEIL1* or *NTHL1*. Genotyping analyses of independently ascertained FC cancer study groups identified multiple carriers with OC harbouring the same variant, which is likely due to common ancestors within the FC population of Quebec (33–35). Overall, carriers of each variant are rare, each accounting for <1%, but collectively 9.6% of 52 familial OC cases with at least two or more OC cases and 1.6% of all 435 sporadic OC cases versus 0.1% of the population-matched controls. It is notable that none of the variants were found in known OC predisposing genes (nccn.org/guidelines/category_2), confirming prior findings from either clinical testing or our WES analyses of this group of cases (24, 43, 47).

Our candidate variants were identified by investigating a curated list of 468 known or putative genes involved in different DNA repair pathways. *ERCC5* is known to be involved in the NER pathway as an endonuclease, but it has been shown that this gene is also involved in the BER (117) and HR (108) pathways. *EXO1* is involved in the HR and MMR pathways as an exonuclease (118, 119). *FANCC* plays a role in the FA pathway as a member of the core complex (120). *NTHL1* and *NEIL1* are DNA glycosylases in the BER pathway (121). Although the role of these genes in conferring risk to hereditary OC requires further investigation with larger cohorts, a recent Australian study of familial and sporadic OC cases reported that there was a statistically significant difference in the frequency of germline LoF variants in the single-stranded DNA repair pathway genes involved in BER, NER and MMR in OC cases versus non-cancer controls (17). On the other hand, homozygous or compound heterozygous PVs in *NTHL1* have been linked to Familial adenomatous polyposis - 3 (MIM: 616415) (122) and most recently a multi-tumour phenotype (123). Homozygous or compound heterozygous PVs in *FANCC* and *ERCC5* are known to be linked to autosomal recessive disorders, Fanconi anemia complementation group C (MIM: 227645) and Xeroderma pigmentosum complementation group G (MIM: 278780), respectively, known to exhibit increased risk to cancer.

Three of our top candidate variants were predicted to be LoF: *NTHL1* c.244C>T; p.Gln82Ter, *EXO1* c.1268-1G>T and *FANCC* c.897G>T; p.Arg299Ser. *NTHL1* c.244C>T; p.Gln82Ter has been independently reported in the literature due to its frequency, while *FANCC* c.897G>T; p.Arg299Ser has been reported only in ClinVar database. The introduction of a termination codon in *NTHL1* p.Gln82Ter is predicted to affect NTHL1 protein production, eliciting its classification as PV in ClinVar and by ACMG guidelines. *EXO1* c.1268-1G>T and *FANCC* c.897G>T; p.Arg299Ser were predicted to affect splicing by all of our selected in silico tools. As RNA was not available from carriers of these variants, we were unable to investigate their effect on the gene transcripts. We applied a stringent criteria for prioritizing missense variants using a selected set of high performance in silico prediction tools (71–74). *ERCC5* c.2556A>G; p.Ile852Met and *NEIL1* c.248G>T; p.Gly83Asp, which have been independently reported in the literature due to their frequencies, were among our top prioritized missense variants. A recent study assessed the performance of 44 in silico tools with 70 tool-threshold combinations in predicting missense variants using a curated dataset of over 9,000 missense variants in five OC and/or BC risk genes that were classified as deleterious or tolerated based on different functional assays (73). Two of the in silico tools that were selected in our analysis, REVEL with a threshold of >0.7 and VEST with a threshold of ≥0.5 prediction scores of a missense variant being deleterious, were shown to have the best performance of 79% and 74%, respectively (73). Moreover, a combination of both tools with these prediction score thresholds boosts the prediction performance up to 81% (73). *NEIL1* c.248G>A; p.Gly83Asp had REVEL and VEST scores of >0.7, while *ERCC5* c.2556A>G; p.Ile852Met had a REVEL score at the threshold of 0.7 and VEST score of 0.9. The biological impact of ERCC5 p.Ile852Met is unknown, though the variant alters a codon in the highly conserved I-Nuclease domain (see **Figure 4**), which may impact ERCC5 endonuclease activity. Whereas, cells expressing NEIL1 p.Gly83Asp have been shown to increase levels of stalled replication forks and double-strand breaks as compared to wild-type NEIL1 (109). For the missense variants identified in the non-FC cases, eight of the nine missense variants were predicted to be deleterious by REVEL (>0.7) and/or VEST (≥0.5). One variant, *NTHL1* c.349C>T; p.Pro117Ser, was found to have a REVEL score of 0.6 and VEST score of 0.5, which is within the intermediate window of prediction scores (REVEL <0.7->0.4; VEST<0.5) where the threshold of predicting missense variants being tolerated is <0.4 (73). It is evident that in silico tools are being developed with increasing improvement in their performance and are useful alternatives to biological modelling of variants for selecting and prioritizing missense candidates for further characterization (73, 124).

We applied stringent criteria to select top candidates for further analyses as it was not feasible to perform WES on all our FC OC study cases. Though selecting for rare variants with MAF ≤0.005 aligns with our hypothesis for identifying candidate moderate- to high-risk variants with the assumption that new candidate genes are transmitted *via* an autosomal dominant mode of inheritance (63, 114, 125, 126), we filtered out our recently reported, LPV *FANCI* c.1813C>T; p.Leu605Phe (24). Notable is that the one family harbouring this *FANCI* variant among the 13 familial cases investigated in this study, did not harbour any of our top candidates. As our strategy selected but not eliminated top candidates, further research is required to determine their relevance to OC risk. Moreover, as we have shown in our studies of predicted missense identified in *RAD51C* and *RAD51D* (43) and *FANCI* (24) in OC cases from the FC population, modeling variants by in cellulo assays would provide further evidence for their relevance in OC biology.

Some of our candidate variants or others in these genes that met our selection criteria were identified in 3.3% of 937 non-FC familial or sporadic OC cases of mostly European ancestry 0-0.004% in gnomAD controls, suggesting that our gene candidates may be relevant in other populations. Though this observation was not unexpected, given that approximately 55% of our non-FC OC cases were from the same Australian study group (17), they are also consistent with our analysis of the Pan-Cancer TCGA OC cases. During the course of this study, a recent report investigated a set of DNA repair genes in 33 different cancers from Pan-Cancer TCGA, and they found that missense variants predicted damaging are statistically enriched in OC cases (127). Moreover, carriers of LoF variants in some of our candidate genes have been reported in HBOC families such as *ERCC5* (128, 129)*, FANCC* (130–133) or *NTHL1* (111, 134, 135) from different populations. Interestingly, LoF variants in *ERCC5* (110), *FANCC* (102) or *NTHL1* (111, 110) have been associated with hereditary BC cases in the context of HBOC families. On the other hand, our literature search did not identify reports of germline PVs in *EXO1* or *NEIL1* in OC, although variants in these genes have been reported in the context of other hereditary cancers such as colorectal cancer (136–138). However, common variants (MAF>1%) in *EXO1* have been associated with OC risk (139).

Although we are limited by sample size, we did not observe any striking clinical characteristics regarding age at diagnosis or histopathology of OC disease in carriers of our candidate variants. The average age at diagnosis with OC in FC variant carriers (average=58; median=60 years) is comparable to that of AUS variant carriers as well as Pan-Cancer – TCGA variant carriers (average=59; median=59 years), which in turn is comparable with that of carriers of *BRCA2* PVs (average=58 years) (41) and the general population (median=63 years) (112, 113). The majority of OC cases harbouring the candidate variants had HGSC (91.1%, 41/45), which is the most common subtype of OC reported in epithelial ovarian cancer (49) and thus is overrepresented in our study groups (56). We did observe three carriers of *EXO1* c.1268-1G>T, *NEIL1* c.248G>A; p.Gly83Asp and *NTHL1* c.244C>T; p.Gln82Ter with mixed histology (serous mixed with endometrioid or unspecified cell type; and endometrioid mixed with mucinous) (see **Table S8**). Interestingly, a survey of our candidate variants in the Ovarian Cancer Association Consortium (OCAC) database (ocac.ccge.medschl.cam.ac.uk/data-projects/, accessed on 15 June 2020), showed that *NEIL1* c.248G>T; p.Gly83Asp was significantly associated with OC overall (odds ratio [OR] = 1.5; p = 0.038), and this association was stronger with the endometrioid subtype (OR = 3.75; p= 0.00008) (see **Table S10**). *NTHL1* c.244C>T; p.Gln82Ter showed a higher OR = 1.5 in clear cell subtype but was not statistically significant (p = 0.36) (see **Table S10**). These observations are interesting as *NTHL1* and *NEIL1* are involved in repairing single stranded-DNA breaks *via* BER pathway. MMR genes as also involved in repairing single stranded-DNA breaks *via* MMR pathway that are associated with conferring an increased risk to the endometrioid and clear cell histological subtypes of OC (7). We could not investigate the other candidate variants *ERCC5* c.2556A>G; p.Ile852Met, *EXO1* c.1268-1G>T and *FANCC* c.897G>T; p.Arg299Ser from this genotyping-based database as they were not represented in the SNP arrays, which could be due to their rarity in the general population.

The role of our candidate genes in the etiology of OC is unknown, though LOH analyses suggest that loss of function of some of our candidate genes in tumour cells may be important in tumourigenesis of OC as has been demonstrated for known OC risk genes (114). We showed loss of the wild-type allele in tumours from carriers of *ERCC5* c.2556A>G, *NEIL1* c.248G>T; p.Gly83Asp or *NTHL1* c.244C>T; p.Gln82Ter. We also showed loss of the wild-type allele in the tumour DNA from the left and the right ovaries from two *NTHL1* c.244C>T carriers with bilateral OC. This suggests the possibility that loss of wild-type allele occurred at an early stage in tumourigenesis (140). However, we observed no LOH in tumour DNA from one carrier each of *ERCC5* or *NEIL1* variants and two carriers of *NTHL1* variant (see **Table S7**). In previous studies we have also demonstrated complete or partial loss of wild-type alleles in tumour DNA from FC carriers of *RAD51C* c.705G>T; p.Lys235Asn (43), *RAD51D* c.620C>T; p.Ser207Leu (43, 44) and *FANCI* c.1813C>T; p.Leu605Phe (24) also from the analyses of RRCancer biobank materials. We also observed no LOH in some of the tumour DNA from OC carriers of our *RAD51C* c.705G>T; p.Lys235AsnAs and *RAD51D* c.620C>T; p.Ser207Leu, as the DNA was extracted post-chemotherapy treatment, suggesting the possibility of stromal cell contamination (43, 44). Indeed, all of the DNA tumours from the four cases analysed in this study were confirmed to be extracted after chemotherapy. Interestingly, our LOH analyses in this study provided evidence for loss of variant allele from other candidate variants *EXO1* c.1268-1G>T and *FANCC* c.897G>T carriers. It is not clear if this is due to contaminating normal tissues as our analyses were not performed on selected tumour cells as HGSC samples are often enriched in tumour cells (24). Srinivasan et al. (141) recently reported that approximately 20% of the 55 investigated cancers, including OC, showed a retention of the wild-type alleles in the high-penetrant genes such as *BRCA1* and *BRCA2* (141). A retention of the *RAD51D* c.620C>T; p.Ser207Leu was also observed in tumour DNA from an OC carrier (44). It is not clear if such cases reflect a reversion of variant to wild type allele as has been shown with *BRCA1* or *BRCA2* carriers in the context of developing resistance to cisplatin or the targeted therapy poly (ADP-ribose) polymerase (PARP) inhibitors (142, 143). Further research is required at the tumour cell level to determine biological impact of variants in the context of wild-type alleles in carriers.

There are several limitations in this original study that should be acknowledged. This study was limited in the sample size of the FC OC families, sporadic cases and cancer-free controls (study phase I and II). The small sample size in our cases and controls did not allow us to estimate the associated risk with OC of any of the candidate variants to support their candidacy as OC predisposing variants/genes (144) as such risk assessment requires thousands of cases and controls (12, 13, 1, 19). However, our highly selected OC families regardless of their status of PVs in *BRCA1* and *BRCA2* are very rare. The proportion of families with two first-degree relatives with OC is estimated to be less than 5%, and less than 1% for those with more than two first-degree relatives with OC (145–147). This study was also limited in lacking of biological samples that were required to confirm the predicted effect on RNA splicing of two of the candidate variants *EXO1* c.1268-1G>T and *FANCC* c.897G>T; p.Arg299Ser. Likewise, we were not able to perform LOH analysis on all OC carriers of the candidate variants as well as genotyping PBL DNA of the other family members to further support the candidacy of our variants. This was due to the fact that the extracted DNA from only PBL and/or tumour specimens were biobanked for almost all of the OC cases. Moreover, histopathological blocks for DNA and/or RNA extraction of these carriers were not available from the respective biobanks as some of the cancer families and cases date back to the early 2000s (see **Table S1**).

In conclusion, our WES and genetic analyses of 468 genes directly or associated with DNA repair pathways in study groups from a genetically defined population identified candidate variants in *ERCC5*, *EXO1*, *FANCC*, *NEIL1* or *NTHL1*. The genetic analyses of these variants and genes in non-FC OC study groups implicate these genes in other populations. Genetic epidemiology of variant carriers and functional assays to assess the biological impact of variant proteins could elucidate the effect of candidate variants to OC risk.

## Data availability statement

The datasets presented in this article are not readily available because sequencing data for familial and sporadic ovarian cancer (OC) cases, CARTaGENE, McGill University-Montreal Neurological Institute (MNI) and Université de Sherbrooke-The Genetics of Glucose Regulation in Gestation and Growth (Gen3G) will be returned to their respective biobanks at the conclusion of our study of OC predisposing genes which is still ongoing. For more information concerning these data contact Patricia N. Tonin at patricia.tonin@mcgill.ca. The data from the analyses of investigation of The Ovarian Cancer Association Consortium (OCAC) and The Genome Aggregation Database (gnomAD) are available from each of these data resource banks.

## Ethics statement

This project has received approval from The McGill University Health Centre (MUHC) REB (MP-37-2019-4783). The patients/participants provided their written informed consent to participate in this study.

## Author contributions

WA and PT conceived and designed the study. WA performed whole exome sequencing and bioinformatics analyses, conducted, and interpreted the genotyping and Sanger sequencing and wrote the initial drafts of this manuscript. TR performed the bioinformatics pipelines. CF aided in bioinformatics analysis. CS aided in collecting and reviewing all clinical pedigrees with associated information. KO performed imputation and provided required information from CARTaGENE genotyping-based data. DS and IC provided required information from in-house sequencing-based Australian ovarian cancer cases. JB and TP provided required information from The Cancer Genomic Atlas (TCGA) sequencing-based breast and ovarian cancer cases. DS, GR and LB provided required information from in-house sequencing-based control data. A-MM-M, DP, WF and ZH provided the study samples and clinical information. CG oversaw the statistical analyses. JR oversaw the WES analysis and bioinformatics pipeline. PT designed and oversaw all aspects of the study. All authors contributed to the article and approved the submitted version.
