# Supplementary material for: Genetic analyses of DNA repair pathway associated genes implicate new candidate cancer predisposing genes in ancestrally defined ovarian cancer cases
Source: Front Oncol. 2023 Mar 8;13:1111191. doi: 10.3389/fonc.2023.1111191 (PMC10030840; doi:10.3389/fonc.2023.1111191)
Supplement: Supplementary file 1 [file DataSheet_1.zip › Supplementary Figure.pdf]

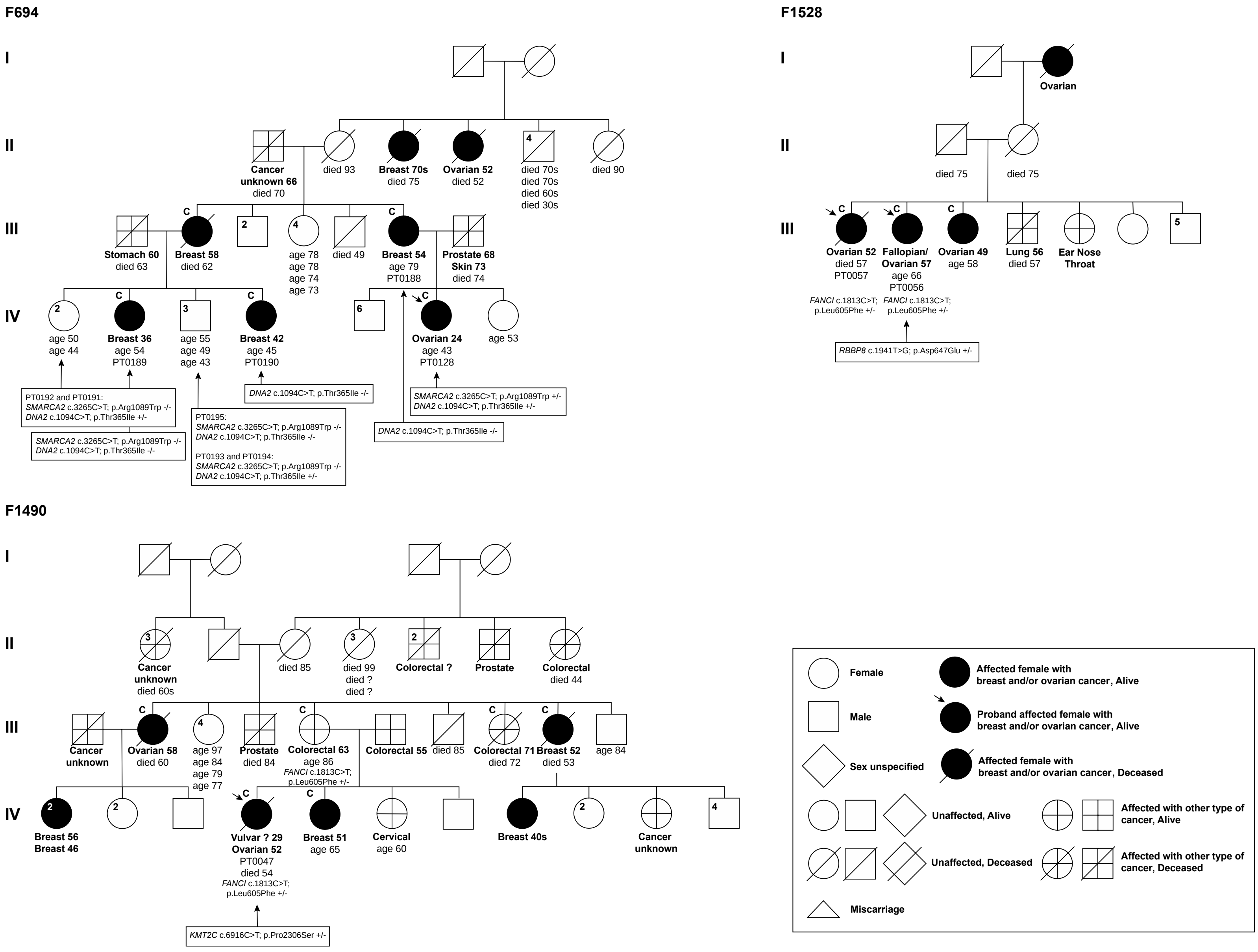
F694
I
II
III
IV
Cancer unknown 66
died 70
died 93
Breast 70s
died 75
Ovarian 52
died 52
4
died 70s
died 70s
died 60s
died 30s
died 90
C
Stomach 60
died 63
Breast 58
died 62
2
4
age 78
age 78
age 74
age 73
died 49
C
Breast 54
age 79
PT0188
Prostate 68
Skin 73
died 74
2
age 50
age 44
C
Breast 36
age 54
PT0189
3
age 55
age 49
age 43
C
Breast 42
age 45
PT0190
6
C
Ovarian 24
age 43
PT0128
age 53
PT0192 and PT0191:
SMARCA2 c.3265C>T; p.Arg1089Trp -/-
DNA2 c.1094C>T; p.Thr365Ile +/-
SMARCA2 c.3265C>T; p.Arg1089Trp -/-
DNA2 c.1094C>T; p.Thr365Ile -/-
PT0195:
SMARCA2 c.3265C>T; p.Arg1089Trp -/-
DNA2 c.1094C>T; p.Thr365Ile -/-
PT0193 and PT0194:
SMARCA2 c.3265C>T; p.Arg1089Trp -/-
DNA2 c.1094C>T; p.Thr365Ile +/-
DNA2 c.1094C>T; p.Thr365Ile -/-
DNA2 c.1094C>T; p.Thr365Ile -/-
SMARCA2 c.3265C>T; p.Arg1089Trp +/-
DNA2 c.1094C>T; p.Thr365Ile +/-
F1528
I
II
III
Ovarian
died 75
died 75
C
Ovarian 52
died 57
PT0057
C
Fallopian/ Ovarian 57
age 66
PT0056
C
Ovarian 49
age 58
Lung 56
died 57
Ear Nose Throat
5
FANCI c.1813C>T; p.Leu605Phe +/-
FANCI c.1813C>T; p.Leu605Phe +/-
RBBP8 c.1941T>G; p.Asp647Glu +/-
F1490
I
II
III
IV
3
Cancer unknown
died 60s
died 85
3
died 99
died ?
died ?
2
Colorectal ?
Prostate
Colorectal
died 44
Cancer unknown
C
Ovarian 58
died 60
4
age 97
age 84
age 79
age 77
Prostate
died 84
C
Colorectal 63
age 86
FANCI c.1813C>T; p.Leu605Phe +/-
Colorectal 55
died 85
C
Colorectal 71
died 72
C
Breast 52
died 53
age 84
2
Breast 56
Breast 46
2
C
Vulvar ? 29
Ovarian 52
PT0047
died 54
FANCI c.1813C>T; p.Leu605Phe +/-
C
Breast 51
age 65
Cervical
age 60
Breast 40s
2
Cancer unknown
4
KMT2C c.6916C>T; p.Pro2306Ser +/-
Female
Male
Sex unspecified
Affected female with breast and/or ovarian cancer, Alive
Proband affected female with breast and/or ovarian cancer, Alive
Affected female with breast and/or ovarian cancer, Deceased
Unaffected, Alive
Affected with other type of cancer, Alive
Unaffected, Deceased
Affected with other type of cancer, Deceased
Miscarriage

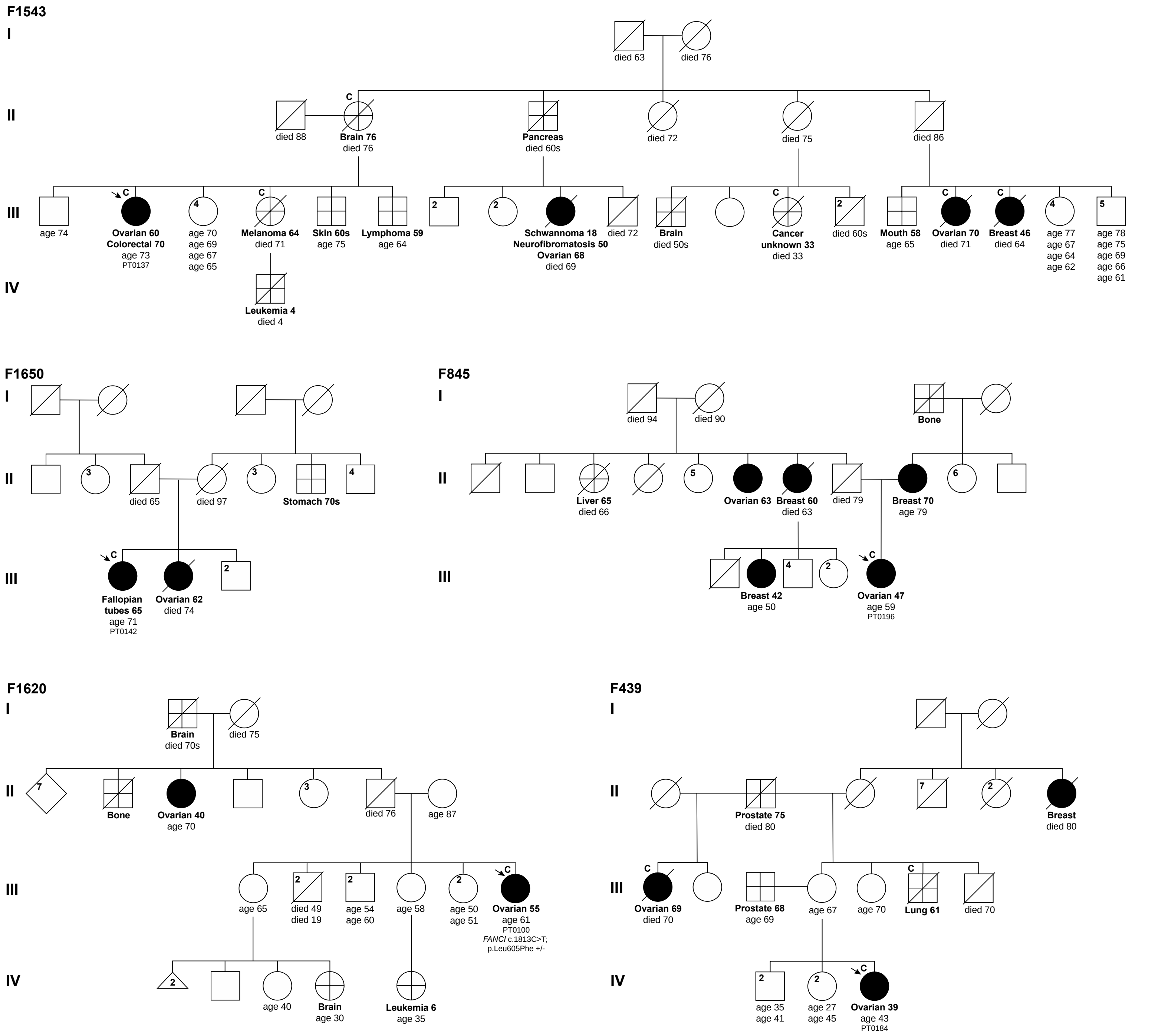
F1543
I
died 63
died 76
II
C
died 88
Brain 76
died 76
Pancreas
died 60s
died 72
died 75
died 86
III
C
C
C
C
age 74
Ovarian 60
Colorectal 70
age 73
PT0137
4
age 70
age 69
age 67
age 65
Melanoma 64
died 71
Skin 60s
age 75
Lymphoma 59
age 64
2
2
Schwannoma 18
Neurofibromatosis 50
Ovarian 68
died 69
died 72
Brain
died 50s
Cancer
unknown 33
died 33
2
died 60s
Mouth 58
age 65
Ovarian 70
died 71
Breast 46
died 64
4
age 77
age 67
age 64
age 62
5
age 78
age 75
age 69
age 66
age 61
IV
Leukemia 4
died 4
F1650
I
II
3
died 65
died 97
3
Stomach 70s
4
III
C
Fallopian
tubes 65
age 71
PT0142
Ovarian 62
died 74
2
F845
I
died 94
died 90
Bone
II
Liver 65
died 66
5
Ovarian 63
Breast 60
died 63
died 79
Breast 70
age 79
6
III
Breast 42
age 50
4
2
C
Ovarian 47
age 59
PT0196
F1620
I
Brain
died 70s
died 75
II
7
Bone
Ovarian 40
age 70
3
died 76
age 87
III
age 65
2
died 49
died 19
2
age 54
age 60
age 58
2
age 50
age 51
C
Ovarian 55
age 61
PT0100
FANCI c.1813C>T;
p.Leu605Phe +/-
IV
2
age 40
Brain
age 30
Leukemia 6
age 35
F439
I
II
Prostate 75
died 80
7
2
Breast
died 80
III
C
Ovarian 69
died 70
Prostate 68
age 69
age 67
age 70
C
Lung 61
died 70
IV
2
age 35
age 41
2
age 27
age 45
C
Ovarian 39
age 43
PT0184

Figure S1. Pedigrees of index ovarian cancer cases each harbouring candidate variants in DNA repair genes in phase I of the study.

Index cases (arrow) subjected to whole exome sequencing analyses (WES), and carrier status of tested index cases and available family members are denoted by plus (carrier) or minus (not a carrier) signs. All carriers were found in a heterozygous state. Age in years is shown at cancer diagnosis and death where applicable. Superscript C denotes histological subtypes that had been confirmed by pathology reports or death certificates at source. Pedigrees of families: F1490, F1620 and F1528 positive for a likely pathogenic variant *FANCI* c.1813C>T; p.Leu605Phe have been reported previously (24).
